# A 300 THz tabletop radar range system with sub-micron distance accuracy

**DOI:** 10.1038/s41598-018-32846-9

**Published:** 2018-09-27

**Authors:** Pierre-Alexandre Blanche, Mark Neifeld, Mingguang Tuo, Hao Xin, N. Peyghambarian

**Affiliations:** 1 College of Optical Sciences, University of Arizona, 1630 E. University Blvd., Tucson, AZ 85721 USA; 2 College of Electrical and Computer Engineering, University of Arizona, 1230 E. Speedway Blvd., Tucson, AZ 85721 USA

## Abstract

We are presenting a compact radar range system with a scale factor of 10^5^. Replacing the radio frequency (RF) by optical wavelength (300 THz), the system easily fit on a tabletop. We used interferometric time-of-flight to reproduce radar ranging measurements. Sub-micron range accuracy was achieved with a 100 fs laser pulse, which correspond to 3 cm for a s-band (3 GHz) radar. We demonstrated the system potential on a simple target, and compared the results with radio frequency measurement using a vector network analyzer. We also present measurement with a more realistic model, a 3D printed reproduction of the USS Arizona battleship, for which a 3D model is extracted from the ranging data. Together with our previous demonstration of radar cross section measurement with a similar system, this report further validates our proposal to use optics to simulate radar properties of complex radio frequency systems.

## Introduction

The RF electromagnetic properties of large and complex structures such as airplanes, ships, and buildings have become increasingly important for a variety of reasons. The radar cross section (RCS) of an object for example defines its range of detection, and even helps for its identification and classification as friend or foe. The scattering of the RF signal from buildings should be taken into account to select the antenna placement and optimize the signal reception for cell phone application.

Since it is not alway convenient to make the measurement in real life: the structure might not be accessible, or not even yet built, radar range has been used as an effective technique to predict the RCS^1–4^. Furthermore, by taking advantage of the scale invariance of the Maxwell’s equations for electromagnetic wave propagation, it is possible to make the radar range much compact than full scale^5^. Indeed, an identical solution is obtained when reducing the size of the object of interest, and increasing the frequency by the same factor. In the past, scale factors applied to radar ranges varied from tens to thousands, allowing to fit the setup in a building, or in a single room^6–8^.

Computer simulations of the RF interaction with objects have taken an increasing place in the understanding of electromagnetic properties. However, for complex and large structures, precise computation is still elusive due to the significant ratio between the volume to be considered, and the size of the wavelength^9,10^.

In a recent publication, our team demonstrated that it was possible to drive the concept of compact radar range much further than it has ever been, by replacing the radio frequency with near infrared light, 100,000 times smaller in wavelength^11^. In this case, the models were fabricated using multiphoton 3D printing technique capable of nanometric resolution. The wavelength was carefully selected to the near IR at 1 micron to leverage the recent advances in photonics for sources and sensors, as well as material availability.

The advantages of using near IR for measuring the RCS is that the setup fits easily on a table top, complex models can be fabricated in a couple of hours, and a 2D array detector can be used to visualize the location of the elements responsible for the scattering signal. This later feature is particularly useful to correct the shape of the structure during the design phase to minimize the RCS.

In the present manuscript, we used a femto-second Ti:Sapphire laser in a time-of-flight interferometric setup to acquire ranging measurement. Ranging measurement is complementary to the RCS information since it defines the signal that would be observed by a radar unit, rather than establishing the signal scattered by the object in all directions (RCS). Additionally, ranging measurement can contain tomographic information (along the axis of wave propagation), that is not present in the RCS.

The system we are presenting has a sub-micron ranging resolution which is comparable to centimeters when scaled to the radar s-band (3 GHz). The measurement, obtained in the optical domain, has been compared to actual radar signal and gave an excellent agreement, demonstrating the validity of our approach. Our setup has the added benefit to be able to reconstruct the 3D object probed by the electromagnetic wave from a stack of 2D images, which gave a visual cue about the target being probed.

## Experimental Section

Radar and Lidar ranging are usually done using a time-of-flight configuration and high speed electronic^12^. In such a radar system, a pulse of electromagnetic energy is emitted by the source, scattered by an object, and the back reflected part of the signal is detected by the receiver. The distance is computed according to the time between emission and reception, divided by the speed of light.

For a typical s-band radar with a 3 GHz frequency and a 10 ns of pulse duration (see for example: FURUNO ELECTRIC CO., LTD, “21” Multi-color High-resolution S-BAND RADAR Models FR-2135S/2165DS”), the pulse FWHM (full width half maximum) is 3 meters. The accuracy on the pulse peak location can be determined to at least 1/10^*th*^ of this value, which gives a range accuracy of about 30 cm (depending on the exact specification of the system)^13^.

Scaling down by a factor 10^5^ to near IR frequency, a 300 THz (1 *μ*m) pulse should have a 100 fs length (10^−3^ s), for a range accuracy of at least 3 *μ*m. Such a short pulse can easily be generated using a mode locked Ti:Sapphire laser system. However, photo-detector with a temporal resolution faster than 100 fs are still under development and very expensive. A better approach to achieve *μ*m resolution is to use homodyne detection in an interferometric time-of-flight configuration.

### Interferometric time-of-flight

**Figure 1:**
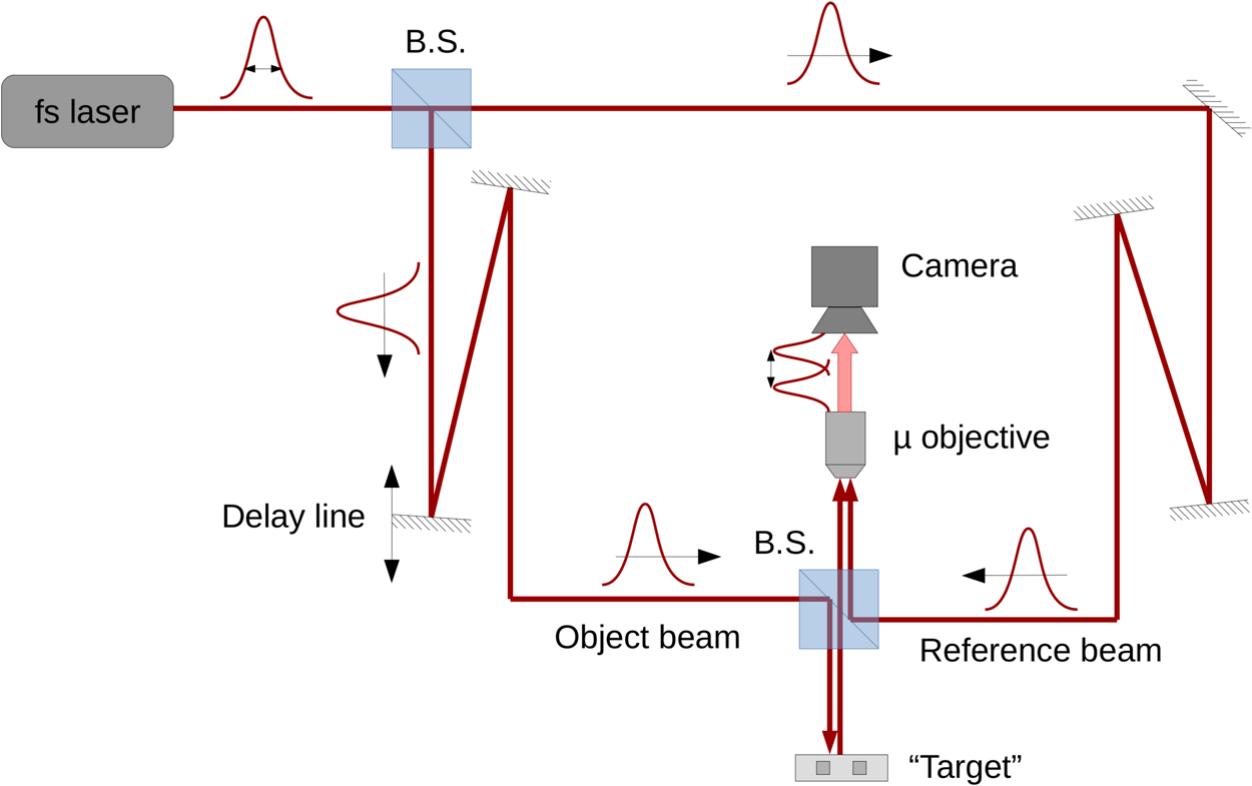
Interferometric time-of-flight setup. See text for description.

An interferometric time-of-flight system is presented in Fig. 1. A coherent pulse of light is emitted by a source and split into two beams by a non-polarizing beam splitter. The reference beam is directly detected by a photo-detector, while the object beam is first sent toward a target, where it is backscattered to the photo-detector. Reference and object beams are coherently combined by another non-polarizing beam-splitter in a Mach-Zehnder interferometer configuration.

In such a system, interference can only be achieved if there is both spatial and temporal superpositions of the pulses. To account for the optical path difference between the two pulses, a delay line is inserted in one arm of the interferometer (object beam in our case). By lengthening or shortening the delay line, it is possible to make interfere the reference beam with part of the object beam pulse that has been backscattered by different sections of the target.

Instead of a single cell photo-detector, we are taking advantage of 2D array detector, and use a camera to see the spatial extend of the interference pattern.

Since the target has been reduced by a factor 10^5^ to account for the same scaling between s-band and IR, a microscope objective is used to magnify the field of view.

### Autocorrelation

**Figure 2:**
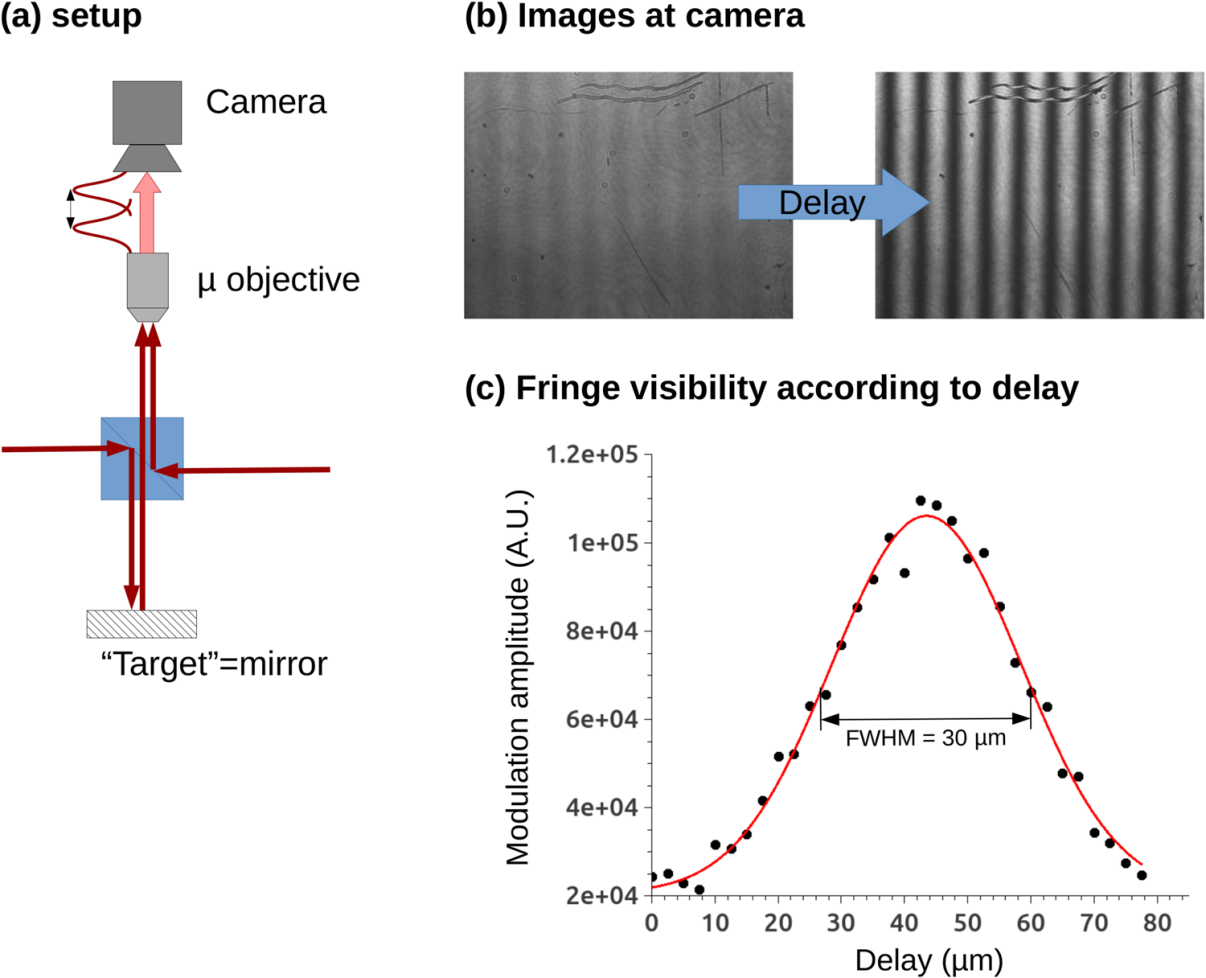
Autocorrelation measurement with a 100 fs pulse. (**a**) Setup schematic. (**b**) Images at the camera for two different delay distances. (**c**) Fringe visibility according to the optical path delay.

We aligned and characterized the setup using a flat mirror located at the target position, and performed an autocorrelation measurement. In such a measurement, presented in Fig. 2, the pulse interfere with itself, and the fringe visibility (intensity modulation between constructive and destructive interferences) is maximum when the optical path delay is canceled. For increasing path delay, the fringe visibility decreases according to the convolution of the pulse temporal profile with itself. This behavior is presented in Fig. 2(c) where the measurement has been interpolated by a Gaussian function with a FWHM of 30 *μ*m. This pulse width value matches the specification of our laser system which delivers 100 fs pulses. The position of the maximum is defined with a precision of ±0.27 *μ*m, which gives the range accuracy of the system.

### Simple target

**Figure 3:**
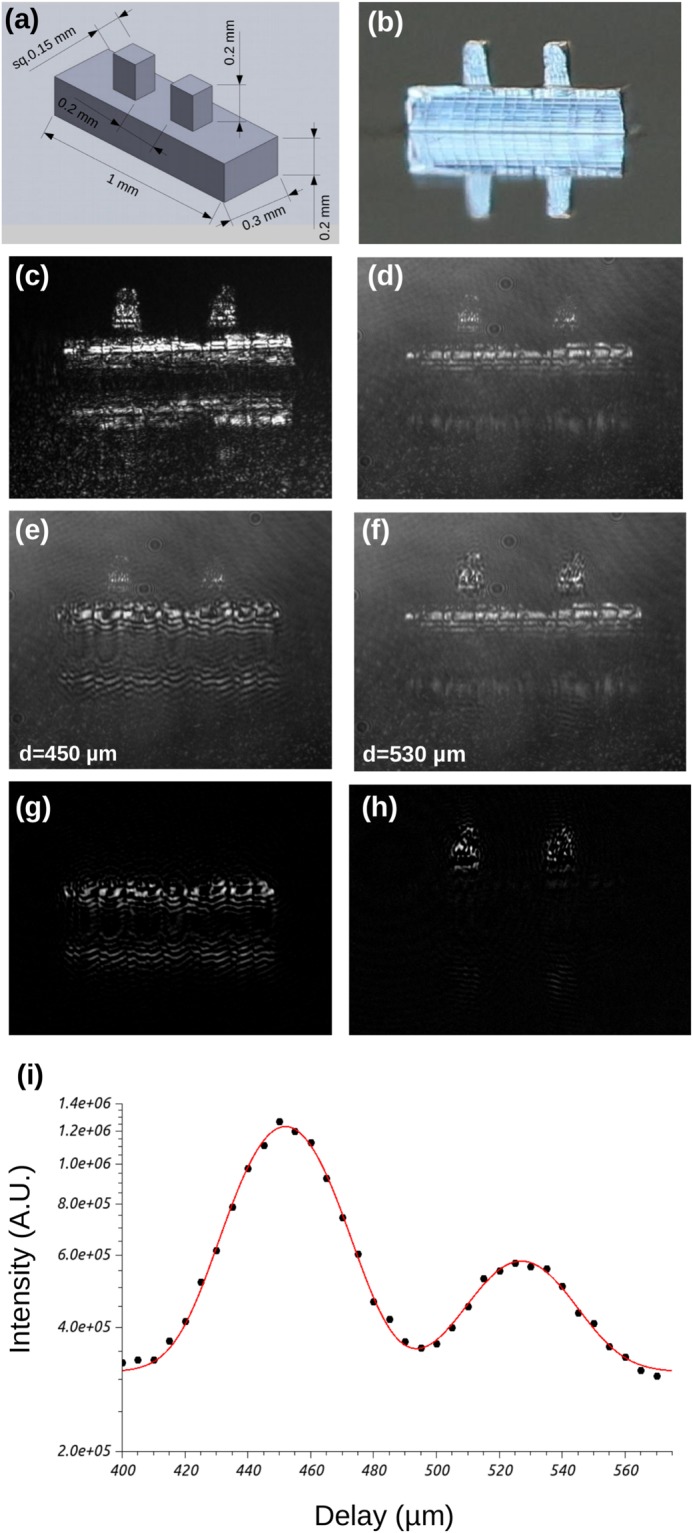
Interferometric time-of-flight on a idealized ship structure. (**a**) Structure dimensions. (**b**) Image of the manufactured structure. (**c**) Structure illuminated with Ti:Sapphire coherent light. (**d**) Object and reference beams turned on (no interference). Beam path difference adjusted to maximize the interference on the hull (**e**), and on the masts (**f**). Image processing to remove the background illumination (**d**), and isolate the interference fringes on the hull (**g**) and the masts (**h**). (**i**) Integration over all pixel intensity according to the optical path delay distance. Plain black dots are measurement, the plain line is an interpolation by the sum of two Gaussians.

We tested our interferometric time-of-flight system with a simple target made of an idealized ship structure. This structure is composed of a large rectangle representing the hull, topped by two smaller rectangles representing two masts. The structure dimension are presented in Fig. 3(a) and have been selected to reflected a 100 meter long ship, scaled by a factor 10^5^. This idealized ship has been micro-machined with a CNC mill out of aluminum, and its photography with an incoherent white light is presented in Fig. 3(b). Tool marks are visible on the side of the different elements, as well as a reflection from the substrate due to the grazing incidence.

Figure 3(c) presents the image observed at the camera when the idealized ship structure is illuminated with the Ti:Sapphire laser beam (without the reference beam). Speckle is visible due to the relatively long coherence length of the laser light. When the reference beam is turned on, the background light level increases (Fig. 3(d)). By changing the beam path delay, interferometric fringes can be observed on the hull part of the model (Fig. 3(e)) or the mast sections (Fig. 3(f)). These two examples are taken from a set of images where the delay was increase by regular steps of 5 *μ*m.

In Fig. 3(e,f), the background light due to the reference beam makes the interference fringes difficult to discern from the rest of the structure. In order to isolate the interference pattern, we processed the images by subtracting the images with ((e) and (f)) and without (d) interference. The result is presented in Fig. 3(g,h) respectively.

Using the set of images where the background has been subtracted, we calculated the intensity of the signal as the sum of the intensity of every pixel. This analysis is particularly useful since it can be compared to the signal retrived from a vector network analyzer (VNA). Figure 3(i) is a plot of the signal intensity according to the beam path delay. The line is an interpolation by the sum of two Gaussians (eq. 1), with parameters given in Table 1.

1 $$y={y}_{0}+A\frac{\sqrt{\mathrm{2/}\pi }}{{w}_{1}}exp[-2{(\frac{x-x{c}_{1}}{{w}_{1}})}^{2}]+B\frac{\sqrt{\mathrm{2/}\pi }}{{w}_{2}}exp[-2{(\frac{x-x{c}_{2}}{{w}_{2}})}^{2}]$$

**Table 1:** Fit parameters for the double Gaussian interpolating the data of Fig. 3(i).

Parameters	First Gaussian	Second Gaussian
Peak amplitude (A.U.):	*A* = 3.45*E* + 07 ± 3.6*E* + 05	*B* = 1.02*E* + 07 ± 3.6*E* + 05
Peak locations (*μ*m):	*xc*_1_ = 451.95 ± 0.13	*xc*_2_ = 527.03 ± 0.48
FWHM (*μ*m):	*w*_1_ = 29.9 ± 0.3	*w*_2_ = 30.4 ± 1.0
Offset (A.U.):	*y*_0_ = 3.12*E* + 05 ± 3.4*E* + 03	

As expected, the ratio between the intensity of the two peaks is equal to the ratio between the surface of the different elements of the model. The hull is 0.2 mm^2^, when the masts have a combined surface of 0.06 mm^2^, which set the ratio at 3.33. The measured peaks have a ratio of 3.45/1.02 = 3.38. which fall within the experimental error.

We compared our results taken in the optical domain, with measurement made with a 90 GHz radio frequency antenna and a VNA. The model used for the RF measurement had a similar geometry to the one presented in Fig. 3(a), but scaled up by the ratio between the frequencies: 300,000/90 = 3,333. Accordingly, the separation between the hull and the masts was 25 cm, and the radio frequency temporal pulse width was 0.3 ns.

**Figure 4:**
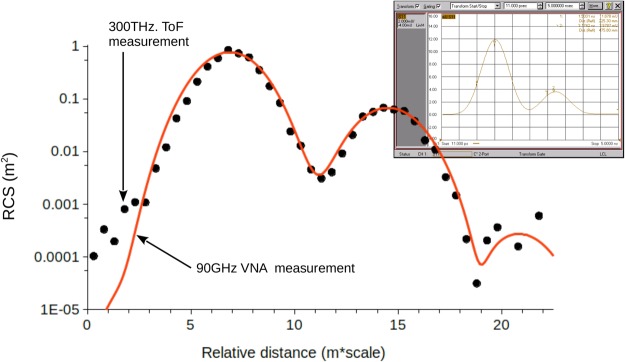
Comparison between the measurements taken in the optics domain at 300 THz (plain black dots), and in the radio frequency domain at 90 GHz (plain line). The structure used in the RF domain is similar to the model presented in Fig. 3(a), scaled according to the ratio between the frequencies (×3,333 for 90 GHz). The measured distance is relative to a full scale model measured at 3 GHz (scale = 1/100,000 for 300 THz, and 1/30 for 90 GHz). Inset: screen capture of the VNA data.

The VNA measurement is presented in Fig. 4 where it is compared to the values obtained in the optical domain. The intensity measurements were converted into RCS values (*σ*) using the theoretical amplitude for a flat rectangular reflector:

2 $$\sigma =4\pi {(\frac{w\times h}{\lambda })}^{2}$$

Where *w* and *h* are the width and height of the rectangle respectively, and *λ* the wavelength of the beam. This formula gives a RCS of *σ* = 0.76 m^2^ for the hull and *σ* = 6.9*E* − 2 m^2^ for the masts, which are indeed the observed values.

**Figure 5:**
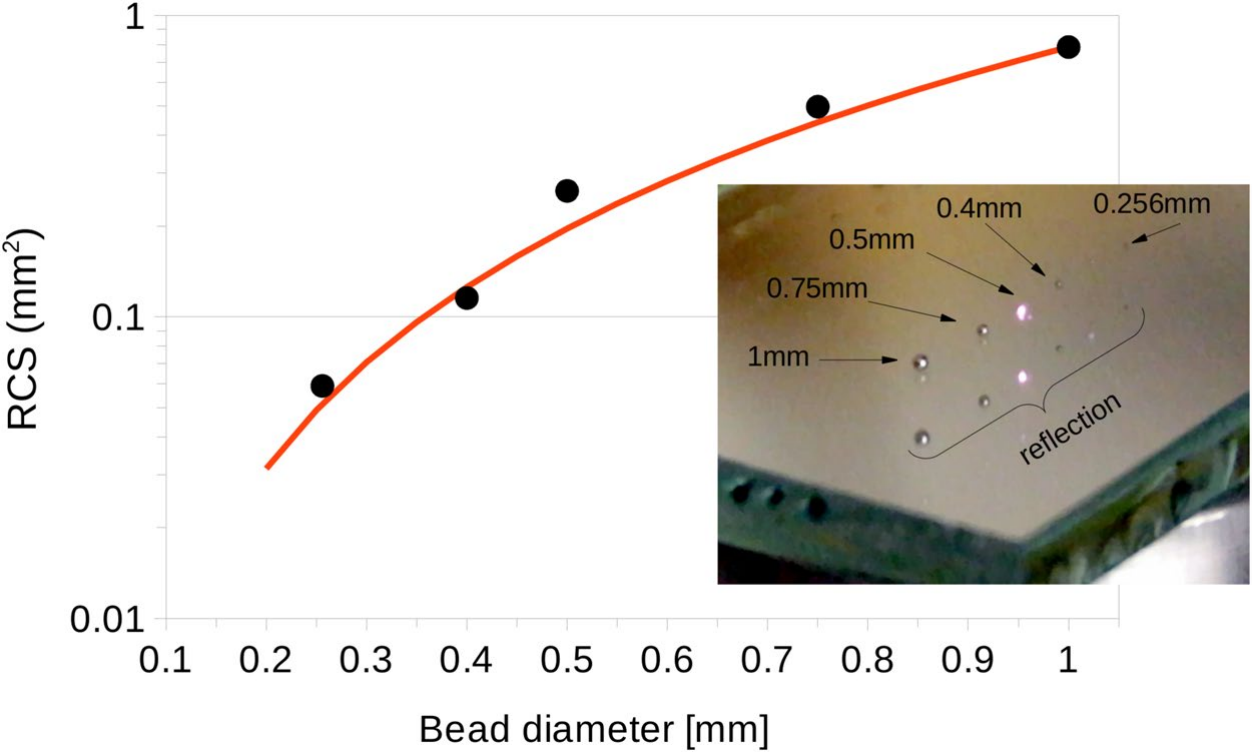
RCS calibration of the optical setup using the reflection from chrome spheres. Data are black dots, theoretical values is red line. Inset: picture of the 5 ball bearing spheres on top of a glass plate. The 0.5 mm diameter sphere is illuminated by the Ti:Sapph laser. Bottom spheres are reflection from the substrate.

Similar to what it is used for radar systems, the optical setup can also be calibrated using reflective spheres. We used chrome ball bearing of various diameters, ranging from 0.256 mm to 1 mm, to measure the RCS and compare it to the theoretical value given by *σ* = *πr*^2^, where *r* is the radius of the sphere. Results are presented in Fig. 5 where good agreement is obtained between experiment and theory.

### Realistic target

In order to test the technique on a more realistic model, we built a replica of the USS Arizona (BB-39). The 3D CAD file of this ship is available in the public domain (See for example: cgtrader.com https://www.cgtrader.com/3d-models/watercraft/military/u-s-s-arizona-8a25a2a9c738ebd76043481a457b62a0, accessed: 04/18/2017), and was used to fabricated a 100,000 scaled replica by 3D printing. The 2 mm long model required only 3 hours to print with the Nanoscribe Professional GT printer, and cost ≈100 USD in machine operation charges. SEM images of the reproduction have been presented in an earlier publication^11^. The entire structure was coated with gold to reproduce the high reflectivity of steel in the radio frequency domain at near IR wavelength (>97%).

**Figure 6:**
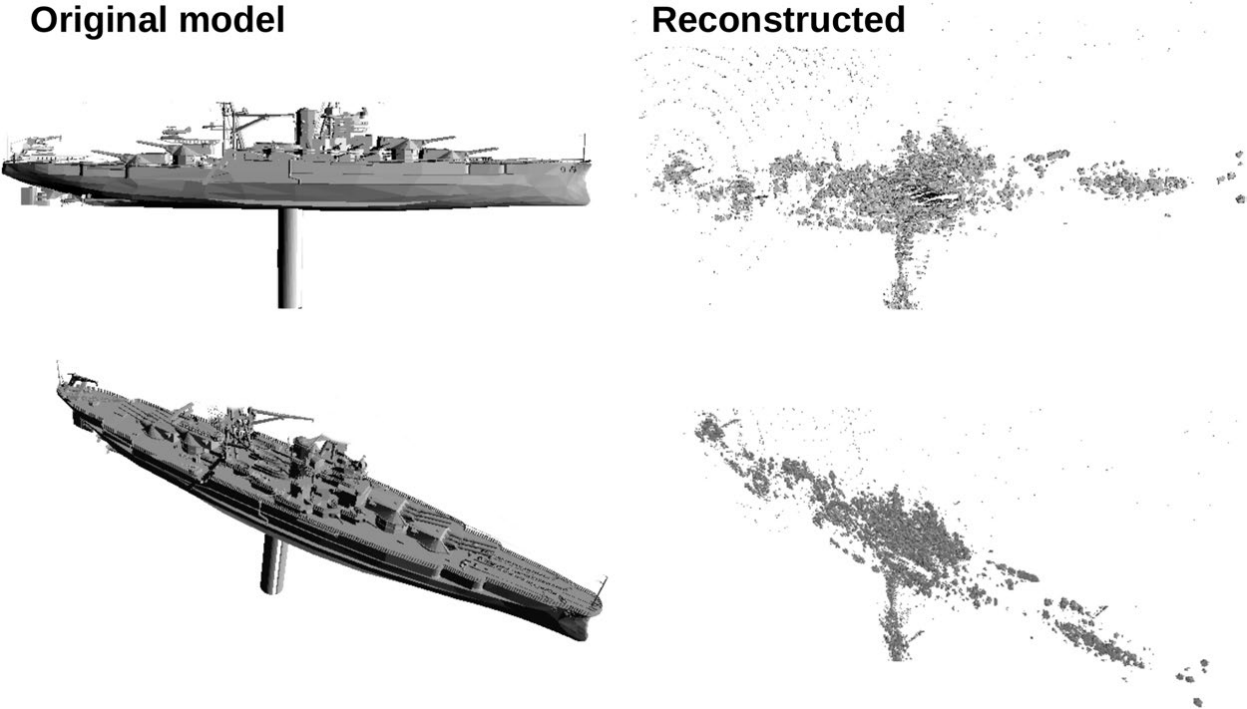
3D model of the USS Arizona. Left panels: original CAD model. Right panels: reconstructed from the interferometric ToF images using a 2 mm long reproduction (1/100,000 scale).

The model was placed in the interferometric time-of-flight setup and 118 images were taken, each separated by 5 *μ*m distance at the delay line. The image were processed to cancel the background as explained previously for the simple target. The stack of image was then imported into the image processing software 3D slicer to generate a 3D model^14^. The result is presented in Fig. 6 where the original CAD model and the reconstructed image from the ranging measurement are presented side by side in two different orientations.

## Discussion and Conclusion

There are different types of noises and artifacts in the reconstructed model presented in Fig. 6. There is some scattered signal in the form of rings coming from strongly reflective elements. Also, some regions are obscured and not visible, either due to shadowing by other elements, or because their reflectivity is bellow the detection threshold. These artifacts are not unique to our system, and are also expected to be observed by a RF radar system.

In a sense, the observed artifacts are not error, but features. It should be noted that our objective is not to measure the target with the best possible resolution, but to demonstrate that optics can be used to simulate RF radar signal. Here we showed that interferometric time-of-flight can be used to reproduce the ranging capability of a radar system, with a setup that easily fit on a tabletop.

Commercial radar systems in the 3 GHz band usually have a range accuracy of about 30 cm (depending of make and model). As presented, our system has a ranging accuracy of ±0.3 *μ*m, which corresponds to 3 cm when scaled up to 3 GHz. This accuracy is a direct property of the pulse width of our laser system (100 fs = 30 *μ*m), and can be increased or decreased using laser pulse shaping to match the specifications of a particular radar system or RF band.

The second limitation to the range accuracy in our setup is the translation stage we used in the beam delay line. The stage has a minimum increment step of 5 *μ*m. However, we showed in our auto-correlation measurement that it was possible to interpolate the fringe visibility information obtained with this increment to obtain a more accurate value of the range (±0.27 *μ*m).

It is possible to dramatically improve the ranging accuracy of the optical setup by using a phase shifting technique. In this case, it is not the modualtion of the interference fringes that is analysed, but their displacement. Using this technique, together with a piezotransducer, a resolution of a fraction of a wavelength (10 nm = 1 mm at 3 GHz) can be achieved^15^. However, considering the present system already has a 10 times better accuracy than commercial radar systems, this extra step does no seem to be needed.

Another source of inaccuracy in the technique we presented is the resolution of the scaled model. This resolution is strongly dependent of the manufacturing technique used to make the replica. The nano 3D printer we used to fabricate the USS Arizona ship has a 3D lateral feature size specification of 150 nm, which corresponds to 15 mm at 3 GHz (20 times better than a s-band radar range accuracy). However, if needed to be, better resolution can be achieved by using lithography (down to 10 nm), or focused ion beam (about 50 nm), in complement to additive manufacturing.

By using 2D detector, our setup has a much better angular resolution (bearing) than a rotating radar system. It is also 2 dimensional since we collect both azimuthal and polar information. This resolution can easily be downgraded by combining several pixels together, up to the entire frame, to simulate the signal that should be received by a specific radar equipment. That is precisely the technique used to compare our data to the RF VNA measurement, which does not have any angular resolution (see Fig. 4).

The use of a 2D detector also helps increase the dynamic range of the system by spreading the signal over a larger surface area than with a single pixel detector. In fact, the maximum dynamic range is multiplied by the number of pixels included in the image field (1280 × 960). However, this maximum value can only be achieved for an object that fills the entire image field. Nevertheless, Fig. 4 shows that the system can achieve at least 40 dB of dynamic range with a real object. It is also possible to improve upon this value of the dynamic range, by combining measurements taken with different laser intensities, reducing the power to avoid saturation, and increasing the intensity to improve the visibility of less reflective elements.

Together with the earlier demonstration that a similar setup is capable to accurately reproduce RCS measurement^11^, this report validates our proposal that optical wavelength can be used in a table-top compact radar range. This new compact radar range can be used to determine the RF signature (RCS) of a structure that cannot be acquired otherwise, either due to its size or complexity. The nano 3D printing technique used to produce the scale object is fast, and economical, which allows to obtain the measurement in a matter of hours instead of months for larger radar ranges. The versatility of the optical setup, both in term of pulse temporal profile and beam geometrical characteristics, means that it can be easily adapted to simulate the signal that will be received by a particular radar system according to its frequency and pulse configuration.

We are now working on the reproduction of different materials permittivity, in order to extend the measurements to dieletric media such as rock, soil, wood, and concrete. This will allow simulating the radar properties of extended scenes such as forest, fjord, or urban environment.

Another aspect of our research is the possibility to add nano-antenna on the model, and measure their emission pattern (gain) *in situ*. This would help to optimize the antenna placement, to avoid shadow, and interference. This capability could find application for the future 5 G wireless communication systems, for which signal accessibility is important^16,17^.
